# The thrombectomy in limb ischemia score (TILI-Score): score proposal and results of an interobserver readability survey

**DOI:** 10.1007/s10554-026-03617-9

**Published:** 2026-02-18

**Authors:** Aleksandra Tuleja, Stephanie Zbinden, Ludovica Ettorre, Maria-Antonela Ruffino, Greicy Heymann, Céline Deslarzes, Tim Sebastian, Anna-Leonie Menges, Sarah Maike Bernhard, Fabrice Noël Helfenstein, Michel Bosiers, Marc Schindewolf

**Affiliations:** 1 Department of Angiology, Swiss Cardiovascular Center, University Hospital Bern, Bern, Switzerland; 2 Department of Angiology, Geneva University Hospital, Geneva, Switzerland; 3 Department of Vascular Surgery and Angiology, Ente Ospedaliero Cantonale, Lugano, Switzerland; 4 Department of Interventional Radiology, Institute of Integrated Diagnostics of Southern Switzerland, Ente Ospedaliero Cantonale, Lugano, Switzerland; 5 Department of Radiology, Geneva University Hospital, Geneva, Switzerland; 6 Department of Vascular Surgery, Lausanne University Hospital, Lausanne, Switzerland; 7 Department of Angiology, University Hospital Zürich, Zürich, Switzerland; 8 Department of Vascular Surgery, University Hospital Zürich, Zürich, Switzerland; 9 Department of Angiology, Vascular Center, Bienna Hospital Center, Bienna, Switzerland; 10 Department of Vascular Surgery, University Hospital Bern, University of Bern, Bern, Switzerland

**Keywords:** Acute limb ischaemia, Thrombectomy, Revascularisation, Peripheral embolization, TILI score, Interobserver agreement

## Abstract

Standardized angiographic endpoints for evaluating treatment success after thrombectomy in acute limb ischemia (ALI) are lacking, limiting comparability across trials and clinical practice. We developed the Thrombectomy in Limb Ischemia (TILI) score, a novel angiographic classification system to systematically assess the technical efficacy of thrombectomy in ALI of embolic origin. The TILI Score was designed through consensus by a Swiss-wide research group of specialists in angiology, vascular surgery, and interventional radiology. It comprises of two components: (1) *lesion recanalisation* (Grades 0–3), and (2) *peripheral embolisation* (Grades a–c, ±p), the latter assessed only if recanalisation is Grade 2 or 3. For the pilot validation, inter-reader reproducibility, 10 expert readers were asked to grade 10 representative post-thrombectomy angiograms after standardized training. Agreement was quantify using percentage agreement, Gwet’s agreement coefficient (AC2; quadratic and ordinal weights), and intra-class correlation coefficients (ICC). Nine readers completed the assessment. For the full composite scale agreement reached 93.6% (95% CI: 91.2–96.1), with substantial agreement reliability (Gwet’s AC2 = 0.74 and 0.72) and ICC of 0.756 (95% CI: 0.449–0.886). For the main recanalisation grade (0–3), agreement was higher: 95.1% (95% CI: 92.1–98.2), with almost perfect agreement reliability (AC2 of 0.875 and 0.862). The TILI Score is the first structured and reproducible tool to classify the technical success of thrombectomy in ALI in patients without preexisting occlusive disease. It demonstrated substantial to almost perfect interobserver agreement among experts and may serve as a standardized endpoint for future thrombectomy trials. Broader clinical validation is necessary to define outcome-relevant thresholds.

**Supplementary Information:**

The online version contains supplementary material available at 10.1007/s10554-026-03617-9.

## Introduction

Recent advances in endovascular thrombectomy have transformed the treatment of acute limb ischaemia (ALI). Modern techniques such as computer assisted aspiration and stent retriever systems are increasingly replacing intra-arterial thrombolysis and open surgery as first-line options. This shift reduces systemic complications of thrombolysis and perioperative risks of surgery. Nevertheless, thrombolysis remains highly effective for fresh thrombus degradation, and open surgical thrombectomy continues to achieve excellent thrombus clearance. Therefore, novel devices must show outcomes comparable to or exceeding those of established therapies to warrant routine clinical adoption and justify its cost [1–4].

While device manufacturers emphasize the potential of novel endovascular systems, large-scale direct comparisons are lacking. A major reason is the absence of a standardised angiographic endpoint for technical success. Beyond clinical outcomes, a clear and reproducible measure of angiographic efficacy is essential for evaluating and comparing treatment strategies. In cardiology and neurology, scoring systems such as the TIMI flow grade and the TICI scale have long offered straightforward, intuitive and consistent evaluations of revascularisation quality. Adopting these systems has not only enabled comparability across studies but has also driven technical innovation and ultimately improved patient outcomes [5–11].

In contrast, ALI presents unique challenges that have so far prevented the development of a comparable tool. ALI often arises from plaque rupture or embolism in the context of pre-existing occlusive disease, with occlusions occurring at multiple arterial levels, including the iliac, femoral, popliteal and crural arteries, each of which has distinct anatomical features and different branching patterns. Moreover, outcomes depend not only on reopening the primary lesion but also on the extent and location of peripheral embolization, which may critically determine limb salvage. To date, trials in ALI have traditionally reported technical success in heterogeneous terms such as ‘restoration of vessel patency’, while relying more on clinical endpoints, including limb salvage, amputation-free survival, mortality, major adverse vascular events, reintervention rates and bleeding complications, particularly in the thrombolysis arms [12–16].

In order to address this unmet need, we developed the Thrombectomy in Limb Ischaemia (TILI) Score. This novel classification system has been designed to evaluate the technical efficacy of revascularisation in acute limb ischaemia (ALI) of embolic orgin. The TILI Score is intended as a reproducible endpoint for thrombectomy trials, regardless of the treatment method used (open surgery or endovascular techniques, with or without thrombolysis). To enable an assessment of the technical efficacy of thrombectomy devices, we focused on patients without pre-existing occlusive disease, as this setting allows the risk of peripheral embolisation associated with a given device to be accurately captured. In this study, we present the score and evaluate the reproducibility of assessments among multidisciplinary vascular experts.

## Methods

### Score development

The Thrombectomy in Limb Ischaemia (TILI) Score was initially designed by the first author and refined through consensus discussions within the Swiss-wide TILI research group. The group included specialists in angiology, vascular surgery, and interventional radiology. All members contributed to defining the framework, terminology, and assessment approach of the score, as well as drafting the present manuscript.

The score was developed to assess the technical success of revascularization in patients without pre-existing peripheral arterial disease, providing a reproducible endpoint for thrombectomy trials, regardless of the treatment modality (open surgery or endovascular techniques).

### Score structure

**Fig. 1:**
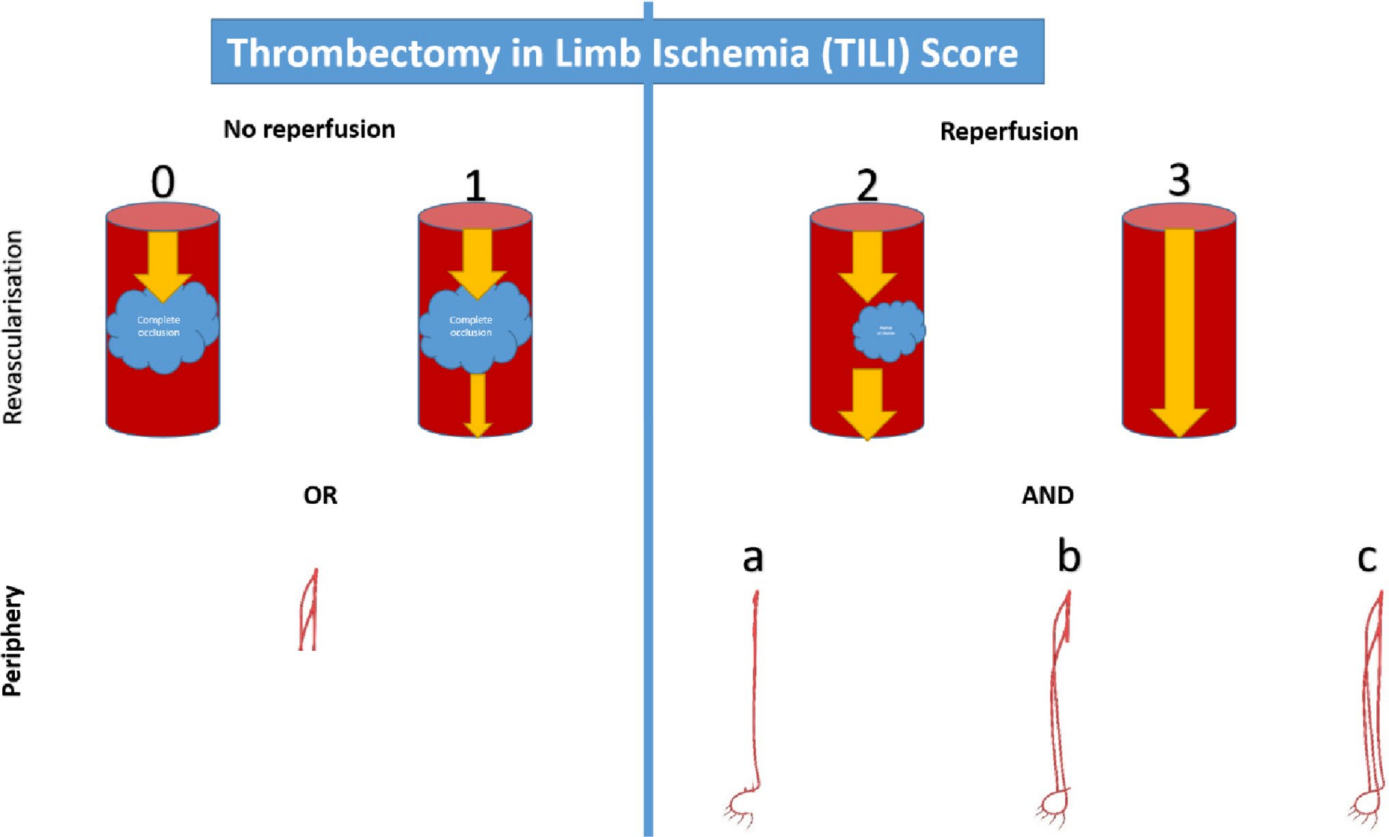
Summary of the Thrombectomy in Limb Ischemia (TILI) Score. The TILI Score consists of two complementary components: (1) Lesion recanalisation (Grades 0–3), assessing reopening of the primary occlusion and subsequent main arterial branches up to the tibioperoneal trunk, and (2) Peripheral embolisation (Grades a–c, ±p), evaluating the extent of distal or side-branch embolisation, assessed only when recanalisation is Grade 2 or 3. Final grades therefore range from 0 to 1 (no effective revascularisation) to 2a/b/c or 3a/b/c, with optional “p” indicating additional proximal embolisation

The TILI Score consists of two complementary components (Fig. 1):

1. **Lesion Recanalisation (Grades 0–3)**: Evaluates reopening of the primary occlusion and all subsequent main arterial branches up to the tibioperoneal trunk, structurally similar to TIMI and TICI grading [17, 18].
  a: **Grade 0**: No recanalisation; no flow distal to the occlusion.
  b: **Grade 1**: No true recanalisation, but some antegrade perfusion (or > 50% residual thrombus).
  c: **Grade 2**: Partial reperfusion with residual thrombus occluding 10–50% of the vessel and antegrade flow through and beyond the lesion.
  d: **Grade 3**: Complete reperfusion with < 10% residual thrombus; fast antegrade flow beyond the occlusion.

**Fig. 2:**
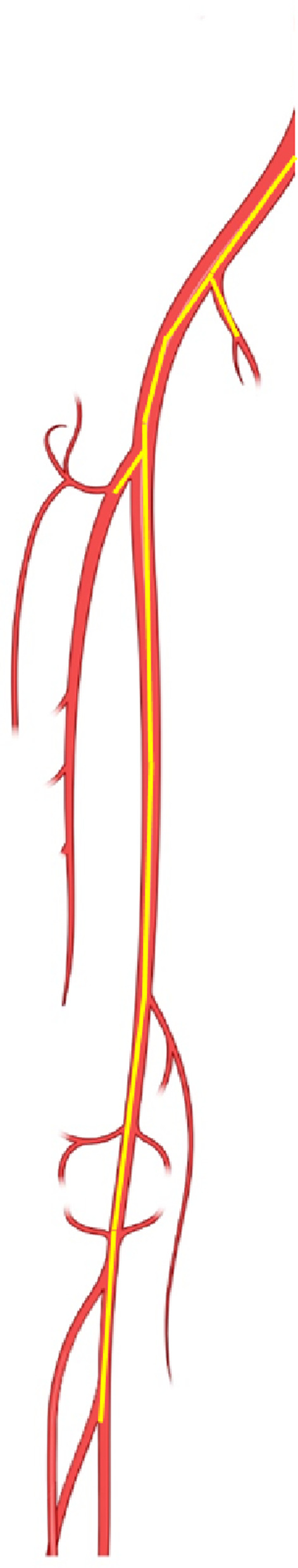
**Assessment of the lesion recanalization (0-3)**: Evaluates reopening of the primary occlusion and all subsequent main arterial branches up to the tibioperoneal trunc as indicated with the yellow line.

Lesion recanalisation is assessed at the site of the primary occlusion and in all subsequent main arterial branches, including the common iliac, internal iliac (main trunk), external iliac, common femoral, superficial femoral, profunda femoris (main trunk), popliteal, and tibioperoneal trunk (highlighted in Fig. 2). This component must be evaluated first in the score. Only if revascularisation is Grade 2 or higher can the second part of the score be assigned. Grades 0 and 1 correspond to the absence of revascularisation.

**Fig. 3:**
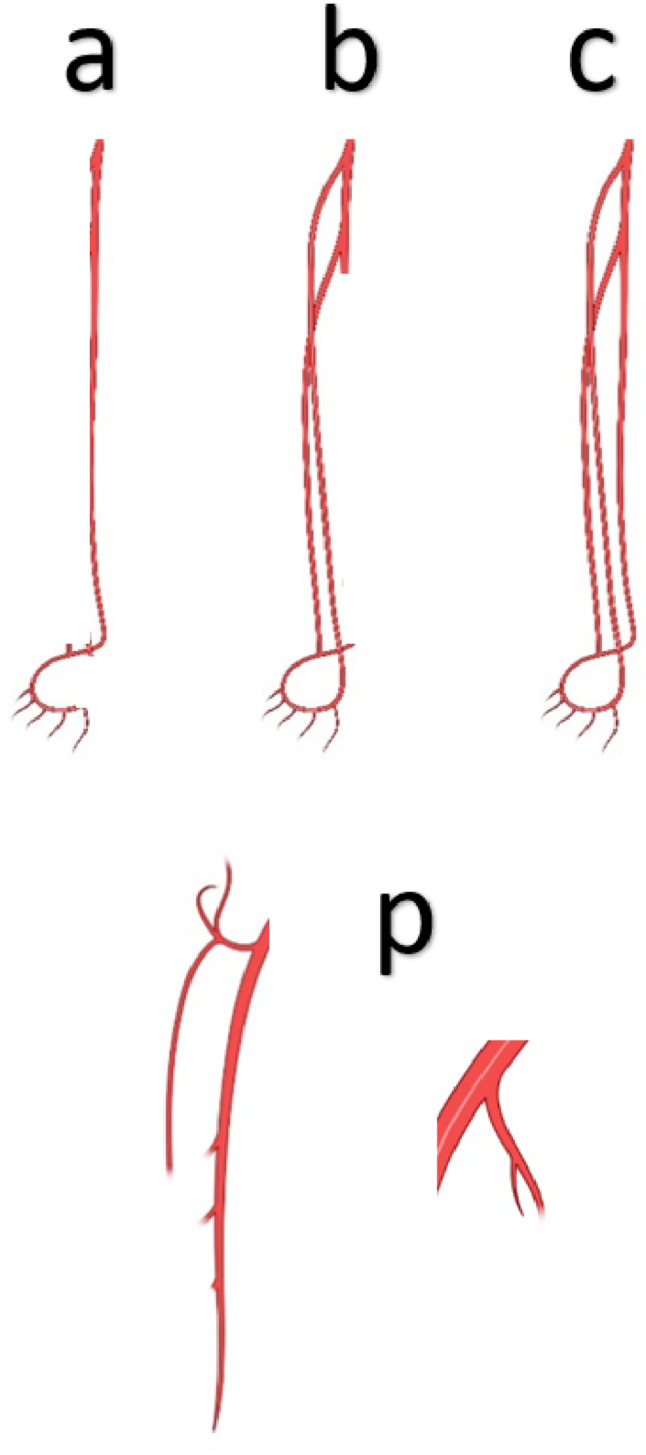
Assessment of peripheral embolization: a: One tibial artery open to the foot; b: Two tibial arteries open (or one tibial + peroneal up to its bifurcation); c: All three crural arteries open with complete plantar arch; p: Embolization in profunda femoris or internal iliac branches beyond its primary division. If all three crural vessels or the main profunda/internal iliac stem are occluded → Grade 0

2. Peripheral Embolisation (Grades a–c, p): Evaluates distal or side-branch embolisation, assessed only when the main recanalisation score is Grade 2 or 3 (Fig. 3).
  a: Single patent crural artery (typically anterior or posterior tibial, but the peroneal artery may qualify if it directly connects to plantar circulation).
  b: Two patent crural arteries (two tibials, or one tibial plus peroneal patent to its bifurcation).
  c: All three crural arteries patent with complete plantar arch.
  p: Embolisation into the profunda femoris or internal iliac arteries beyond the first bifurcation/trifurcation.

If all three crural arteries or the main stem of the profunda femoris/internal iliac remain occluded, the outcome is classified as Grade 0 (no revascularisation).

## Scaling approach

**Fig. 4:**
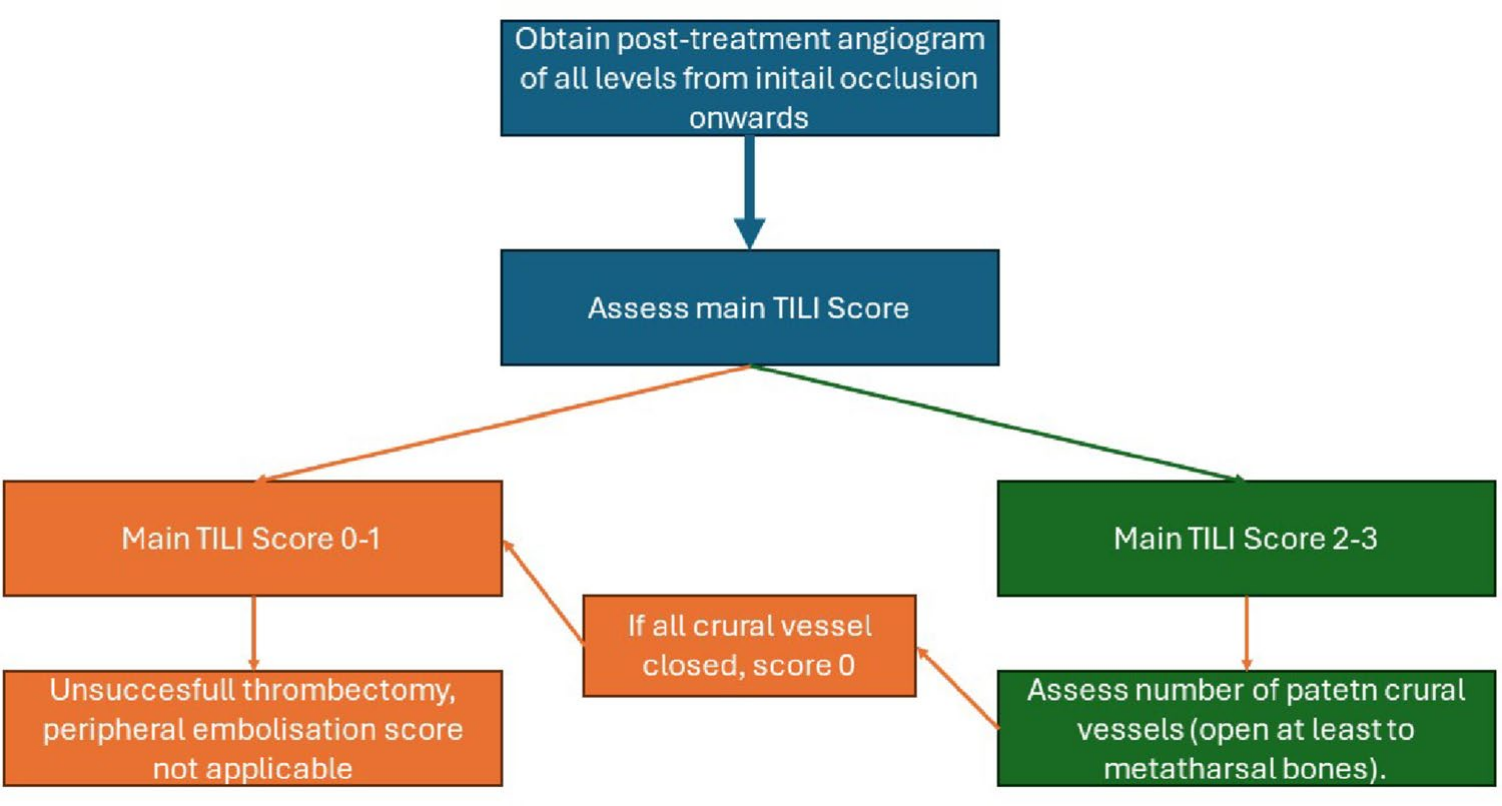
Flow chart on scoring approach

Grading is performed in two steps (Fig. 4):

1. Assign the main recanalisation score (0–3).
2. If Grade 2 or 3 is achieved, assign a peripheral embolisation score (a–c, ±p).

Thus, possible grades are: 0, 1, 2a/b/c ± p, or 3a/b/c ± p. Grades 0 and 1 indicate unsuccessful revascularisation. The a–c scale must always be assigned, while “p” is only used if embolisation involves the internal iliac artery or the profunda femoris artery.

Special considerations include:

- **Tibioperoneal trunk occlusions**: If the anterior tibial artery remains patent but the tibioperoneal trunk remains occluded, the score is Grade 0. If at least one distal branch is open after successful recanalisation, embolisation is graded as usual (a–c).
- **Shifted thrombus**: If the thrombus is displaced distally (e.g., from the femoral bifurcation to the popliteal artery) without complete recanalisation of downstream vessels, the case remains Grade 0 or 1.
- **Crural vessel patency**: Functional foot perfusion determines the peripheral embolisation grade. If only the peroneal artery is patent without a visible pedal arch, this is scored as Grade 0. Small emboli causing < 50% luminal narrowing without hindrance of antegrade flow are considered patent.
- **Pedal arch**: The pedal arch or pedal artery is considered patent if it extends to the level of the metatarsal bones or demonstrates its peripheral branches. (A patent arch may permit concurrent flow, which explains why it is not always visualized following revascularisation.)

### Rationale for the graded scoring approach

The scoring system was designed to be intuitive, easy to apply, and precise enough to convey the essential outcome of revascularization without additional descriptive text. It aims to capture clinically meaningful nuances of revascularization success in limbs without preexisting arterial disease, where anatomical clarity allows reliable assessment of inflow, outflow, and distal runoff.

A two-component structure was chosen because procedural success in acute embolic limb ischemia depends on two distinct elements:

1. Recanalization of the major inflow and outflow vessels, and.
2. The degree of distal embolization affecting crural runoff.

Addressing these separately provides clearer information than a single composite grade.

For the primary inflow/outflow score, the familiar 0–3 grading follows the structure of established reperfusion systems such as TIMI and TICI, which are widely adopted because they support rapid communication and reproducible interpretation. The mTICI refinements (2a/2b/2c) demonstrate how graded distinctions can add meaningful nuance—an approach mirrored in the peripheral embolization component of the TILI score. The lower-limb adaptation required inclusion of the deep femoral artery, the internal iliac artery, and the length of the lesion down to the tibioperoneal bifurcation, as these segments are critical determinants of successful reperfusion.

Segment prioritization within the main score was clinically guided. The deep femoral artery was included because of its major contribution to long-term limb viability through its collateral network. The internal iliac artery, although generally well collateralized, remains a major proximal branch and an important technical target; failure to recanalize it represents a relevant procedural limitation and was therefore integrated into the primary score. Minor branches are assessed only to their first-order divisions, as patency beyond this level does not typically alter perfusion outcomes in non-atherosclerotic limbs. The tibioperoneal trunk was included because it is a frequent site of cardioembolic occlusion and a key determinant of distal runoff.

The peripheral embolization sub-score uses an a/b/c categorization to reflect increasing involvement of tibial and peroneal arteries. This system remains simple—especially given that the lower limb has only three crural arteries—and is conceptually aligned with the graded refinements used in TICI. Grading distal embolization rather than using a binary yes/no assessment provides better insight into device performance and the specific hazard of distal embolization. Tibial arteries were prioritized because they contribute most to pedal perfusion in limbs without chronic disease, while the score remains flexible for anatomical variants dominated by the peroneal artery. The purpose of this sub-score is to represent the adequacy of pedal perfusion after the procedure, which will be correlated with limb outcomes in future work.

Although this study focuses on inter-reader agreement, the broader rationale for a dedicated scoring system lies in the distinct pathophysiology of acute embolic limb ischemia, which differs substantially from acute-on-chronic presentations. A future score for acute-on-chronic disease will require adjustments—such as different prioritization of crural arteries, incorporation of preexisting occlusions, and reduced relevance of the internal iliac artery—but will follow the same structural logic. Establishing a clear and interpretable score for purely embolic cases is an essential first step toward that broader framework.

### Interobserver readability study

To assess inter-reader agreement, a survey evaluating the TILI Score was conducted using 10 representative post-thrombectomy angiograms of the lower extremities (Supplemental Material 1). The angiograms were evaluated by 10 experts in vascular interventions, defined as board-certified vascular specialists (interventional radiologists, angiologists, and vascular surgeons) with at least 5 years of experience in lower-extremity endovascular procedures and routine involvement in catheter-directed thrombolysis, mechanical thrombectomy, and diagnostic angiography. Each expert performed a high volume of limb revascularization procedures annually and was selected based on demonstrated expertise in acute and chronic lower-limb ischemia (Supplemental Material 2).

Before rating, all readers watched an explanatory video and had access to a presentation describing the score [19]. In the survey, each case was presented with the site of the primary occlusion and anonymized final angiographic images (Supplemental Material 1), and the survey was constructed so that each case had to be scored in order to proceed, preventing incomplete responses. Reviewers independently graded each case using the TILI scoring system, applying a 4-point ordinal scale (0–3, with “not applicable” as an option) combined with categorical modifiers (a, b, c, p, or “not applicable”), resulting in a possible set of ordinal outcomes: 0, 1, 2a, 2b, 2c, 3a, 3b, 3c (from worst to best outcome). A structured, moderated online discussion was conducted after the survey to address points of disagreement and ensure a uniform understanding of the scoring principles; although not a formal Delphi process, this approach followed the core principles of iterative feedback and consensus building.

For statistical analysis, the “p” modifier was excluded because it was required in only one case. Inter-reader agreement was evaluated using Gwet’s agreement coefficient for 3 or more readers (AC2; with 95% confidence interval) with quadratic or ordinal weighting to account for the ordinal nature of the TILI score [20–22]. Agreement strength was interpreted against the Landis and Koch benchmark (1977) [23]. Assuming the TILI score was linear and continuous, we further computed the intra-class correlation coefficient (ICC; with 95% confidence interval), also known as repeatability, according to Nakagawa and Schielzeth (2010) [24].

We also assessed inter-reader agreement on the main scale of the TILI score (0–3; corresponding to the degree of revascularization, as described in the TILI score above. The statistical analysis of the agreement was identical to the analysis the full score with inter-reader agreement assessed using Gwet’s AC2 (with 95% confidence interval) with quadratic or ordinal weighting and its strength was evaluated according to Landis and Koch. All analyses were performed using R version 4.4.1 (2024-06-14), using the R-packages irrCAC R [20–22], and prtR [25].

## Results

**Table 1:** Results of interreader agreement of the TILI score survey (ICC intra-class correlation coefficient)

Scale	Metric	Value	95% CI	Interpretation of agreement
Full scale (0–7)	Percentage agreement	93.6%	(91.2, 96.1)	—
	Gwet’s AC2 (quadratic weights)	0.742	(0.618, 0.865)	Substantial [23]
	Gwet’s AC2 (ordinal weights)	0.720	(0.598, 0.842)	Substantial [23]
	ICC	0.756	(0.449, 0.886)	—
Main grade (0–3)	Percentage agreement	95.1%	(92.1, 98.2)	—
	Gwet’s AC2 (quadratic weights)	0.875	(0.772, 0.977)	Almost perfect [23]
	Gwet’s AC2 (ordinal weights)	0.862	(0.758, 0.967)	Almost perfect [23]

Of the 10 invited readers, 9 completed the 10-example survey. Using the entire scale (0, 1, 2a, 2b, 2c, 3a, 3b, 3c converted into 0–7 scale), the overall percentage agreement was 93.6% (95% CI 91.2, 96.1). The inter-reader coefficient of agreement according to Gwet (Gwet’s AC2) for the entire scale using quadratic weights was 0.742 (95% CI 0.618, 0.865). Agreement was “substantial” according to Landis and Koch. With ordinal weights, agreement was also substantial, with Gwet’s AC2 of 0.720 (95% CI 0.598, 0.842). The intra-class correlation coefficient (ICC), assuming the scale was linear and continuous from 0 to 7, was very similar to AC2, with an ICC of 0.756 (95% CI 0.449, 0.886).

Agreement among readers when evaluating the revascularization grade on the 0–3 scale alone was even higher, with a percentage agreement of 95.1% (95% CI 92.1, 98.2). Gwet’s AC2 with quadratic weights was 0.875 (95% CI 0.772, 0.977), corresponding to “almost perfect” agreement according to Landis and Koch (1977). Using ordinal weights, agreement was similar, with AC2 of 0.862 (95% CI 0.758, 0.967), again reaching “almost perfect” agreement according to Landis and Koch (1977) (Table 1).

A descriptive review of scoring discrepancies (Supplementary Material 3) shows that most disagreements occurred in the peripheral embolization component of the TILI Score. In several cases—most prominently Case 4—the distally opacified peroneal artery was interpreted differently by readers: although it filled retrogradely, its origin remained occluded, which correctly corresponds to 3b rather than 3c. Additional disagreement arose in Case 6, where underlying PAD complicated application of the score, and in Case 9, a scenario of primary tibioperoneal trunk occlusion in which the post-thrombectomy appearance remained essentially obstructed, yielding a correct score of 1. These discrepancies were primarily attributable to the use of static end-of-procedure images rather than full angiographic runs, which limited the assessment of antegrade flow and subtle peripheral perfusion, particularly in small-caliber crural vessels.

Across all cases, uncertainty around peroneal artery patency accounted for the majority of disagreements, with discordance reaching 40–50% in the most ambiguous cases. In contrast, patency of the anterior and posterior tibial arteries was consistently identified with markedly higher agreement (typically ≥ 80–90%, depending on the case).

Agreement in extreme categories was nearly complete. Persistent occlusion (grade 0) and full reperfusion without peripheral embolization (grade 3c) showed almost unanimous concordance (Cases 1, 3, and 8). Importantly, there was virtually no disagreement when distinguishing poor outcomes (grades 0–1) from successful reperfusion (grades 2–3), the most clinically meaningful boundary. Remaining discrepancies were therefore confined to the fine granularity of peripheral embolization subgrades and did not affect the major clinical distinction of reperfused versus non-reperfused limbs.

Raw scoring data for all observers across all ten cases are provided in Supplementary Material 3, case-by-case angiographic material is shown in Supplementary Material 1, and reader expertise is detailed in Supplementary Material 2.

## Discussion

We present and validate the Thrombectomy in Limb Ischemia (TILI) Score, the first standardized tool designed to classify the technical success of revascularization in ALI. Among expert readers, agreement in scale evaluation ranged from substantial to almost perfect. The scale can be reliably applied to evaluate post-thrombectomy angiograms in patients without pre-existing lower extremity occlusive disease and is intended as a reproducible endpoint for thrombectomy trials, independent of treatment modality. By providing an objective measure of angiographic success, the TILI Score addresses a significant gap in ALI research, where technical outcomes have traditionally been described in heterogeneous terms.

The TILI Score follows the tradition of established revascularization grading systems, such as the TIMI flow grade in cardiology and the TICI scale in neurointervention and perfoms same or better than those considering interreader agreement [26, 27]. Both of these scores have been instrumental in standardizing reporting, enabling meaningful comparison between devices, and guiding technical refinements that ultimately improved patient outcomes [28–31] Acute limb ischemia research has so far lacked a comparable framework, with studies relying on heterogeneous definitions of technical success and diverse clinical endpoints. By providing a simple and reproducible angiographic classification, the TILI Score represents a step toward similar standardization in the vascular field. Its adoption in future trials could facilitate more consistent evaluation of thrombectomy devices, allow head-to-head comparisons, and ultimately support the integration of technical outcomes with patient-centred measures such as limb salvage and amputation-free survival [6–11, 15, 17, 18].

The TILI score is not intended to replace clinical endpoints but to complement them and provide an angiographic based reproducible assessment of treatment efficacy. Integrating the TILI score in complement to established clinical outcomes in future trials could help clarify the relationship between technical success and long-term clinical outcomes, and support quality and cost-effectiveness analyses in ALI management. In the future, the score must be adapted for patients with chronic occlusive disease, thereby broadening its clinical applicability.

Evidence linking the degree of technical revascularization to clinical outcomes in acute embolic limb ischemia remains limited. Most published studies describe procedural success qualitatively, without applying standardized reperfusion scales or correlating angiographic findings with limb salvage or functional outcomes. Among the few that report results using a scoring system, most adopt the TIMI flow classification for peripheral lesion recanalization, but definitions of “success” vary considerably. Baumann et al. and Fluck et al. define technical success as either an improvement of more than one TIMI grade or achieving an absolute TIMI grade ≥ 2, whereas De Donato et al. modify TIMI into the TIPI Score (Thrombo-aspiration in Peripheral Ischaemia) and classify TIPI 2–3 as successful reperfusion, achieved in 87% of treated cases. Additional technical criteria—such as ensuring patency of at least one crural vessel—were only explicitly specified in the study by Fluck [32–34].

Collectively, these studies demonstrate that TIMI-based peripheral scoring has been used primarily as a descriptive angiographic endpoint, particularly in trials of aspiration systems such as Penumbra/Indigo, but without proven relevance to limb outcomes. None of these studies showed a consistent or validated correlation between TIMI (or TIPI) grade and limb salvage, reintervention, amputation-free survival, or mortality. The TILI Score builds on the general concept of standardized technical grading but is specifically designed for the anatomical and clinical priorities of lower-limb embolic ischemia, including structured assessment of peripheral embolization.

Our experience from the 10 cases included in the inter-reader agreement analysis suggests that TILI grades 0 or 1 were consistently associated with early limb loss or the need for re-intervention, indicating a clinically meaningful relationship between poor angiographic outcome and adverse events. However, this small sample does not allow differentiation of outcomes across higher grades (e.g., 2a, 3a, 3b), nor does it permit conclusions regarding long-term limb salvage or functional recovery.

A limitation of the current TILI Score is that it was developed for embolic occlusions without pre-existing occlusive disease, and therefore is not directly applicable to the largest subgroup of ALI patients with acute-on-chronic ischemia. This intentional restriction was chosen to evaluate the score under the purest anatomical conditions. Importantly, the 0–3 recanalization component can already be applied more broadly, and work is ongoing to adapt the full score to acute-on-chronic ischemia. As this is a pilot validation study based on 10 representative cases, the findings should be interpreted accordingly; a larger, prospectively collected dataset is planned to further validate the scoring system.

Additional technical limitations relate to the survey format and potential selection bias. The survey relied on static images rather than full angiographic runs, which limits assessment of flow dynamics and, given the small caliber of crural vessels and potential differences in projection angles used in other centers, may complicate interpretation of tibial and peroneal patency. This limitation likely led to an *underestimation* of agreement and therefore does not threaten the validity of our findings. A further limitation is that the first author intentionally selected cases to represent the full range of possible score outcomes (0–3, a–c), whereas in clinical practice thrombectomy typically results in high-grade reperfusion (e.g., 3c). This again tends to underestimate interobserver agreement, as complete reperfusion cases showed very high concordance (e.g. case 1 in the survey). Finally, agreement was assessed among experts in the field, which may limit generalizability to less experienced providers. However, because the TILI Score is intended to be applied by trained physicians interpreting their own angiograms, a certain degree of experience is presumed. The conceptual framework aligns closely with the established TIMI/TICI grading systems, which should support wider adoption, although the clinical relevance of distinctions above grade 2a will need to be determined in future outcome studies.

## Conclusion

In conclusion, the TILI Score provides a structured and reproducible method for assessing the technical success of thrombectomy in acute limb ischemia. Tested among experienced readers, it demonstrated substantial to almost perfect agreement and fills a significant gap by offering an objective angiographic endpoint for future clinical trials. While its current application is limited to embolic occlusions without underlying occlusive disease, further refinement and validation in larger, more diverse cohorts will be essential to extend its use in patients with acute-on-chronic ischemia. The adoption of the TILI Score in upcoming studies may not only improve comparability between devices and techniques but also help link procedural outcomes with long-term clinical endpoints, ultimately supporting better standardisation and optimisation of care in limb ischemia management.

## Supplementary Information

Below is the link to the electronic supplementary material.

Supplementary Material 1

Supplementary Material 2

Supplementary Material 3

## Data Availability

Dataset is provided in the Supplementary Material 3 .
