# Supplementary material for: The thrombectomy in limb ischemia score (TILI-Score): score proposal and results of an interobserver readability survey
Source: Int J Cardiovasc Imaging. 2026 Feb 18;42(4):711–20. doi: 10.1007/s10554-026-03617-9 (PMC13053501; doi:10.1007/s10554-026-03617-9)
Supplement: Supplementary file 1 — Supplementary Material 1 [file 10554_2026_3617_MOESM1_ESM.pdf]

Interobserver readibility survey for validating TILI Score

- Used as a google form with registered answers
- Under each answer there was a TILI Main Score, TILI embolization score and comment field.
- Explanatory video for scoring: https://share.descript.com/view/vaY5PBTQOQp

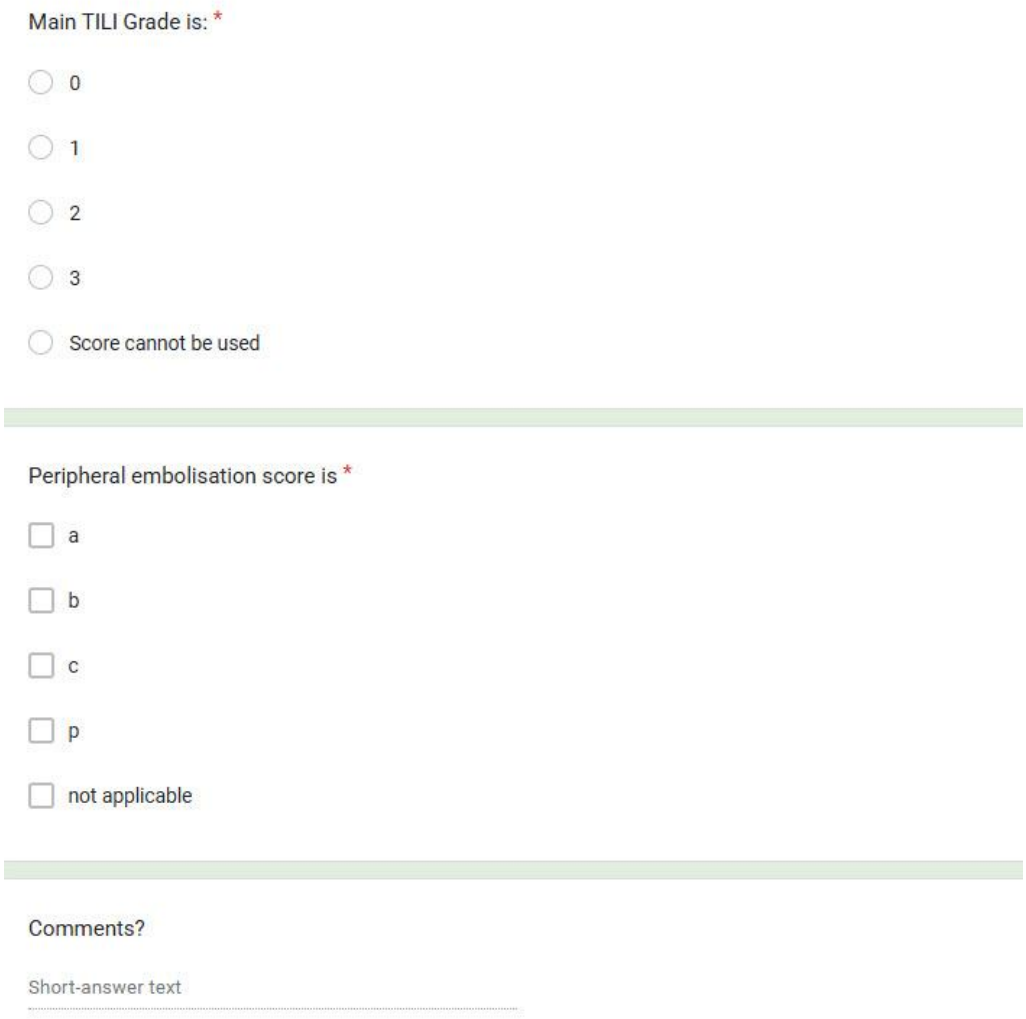

Main TILI Grade is: *

- 0
- 1
- 2
- 3
- Score cannot be used

Peripheral embolisation score is *

- a
- b
- c
- p
- not applicable

Comments?

Short-answer text

Case 1

Patient presented with an occlusion of tibulofibular trunc. The post thrombectomy angiography showed following results:

Images after thrombectomy of tibulofibular trunc

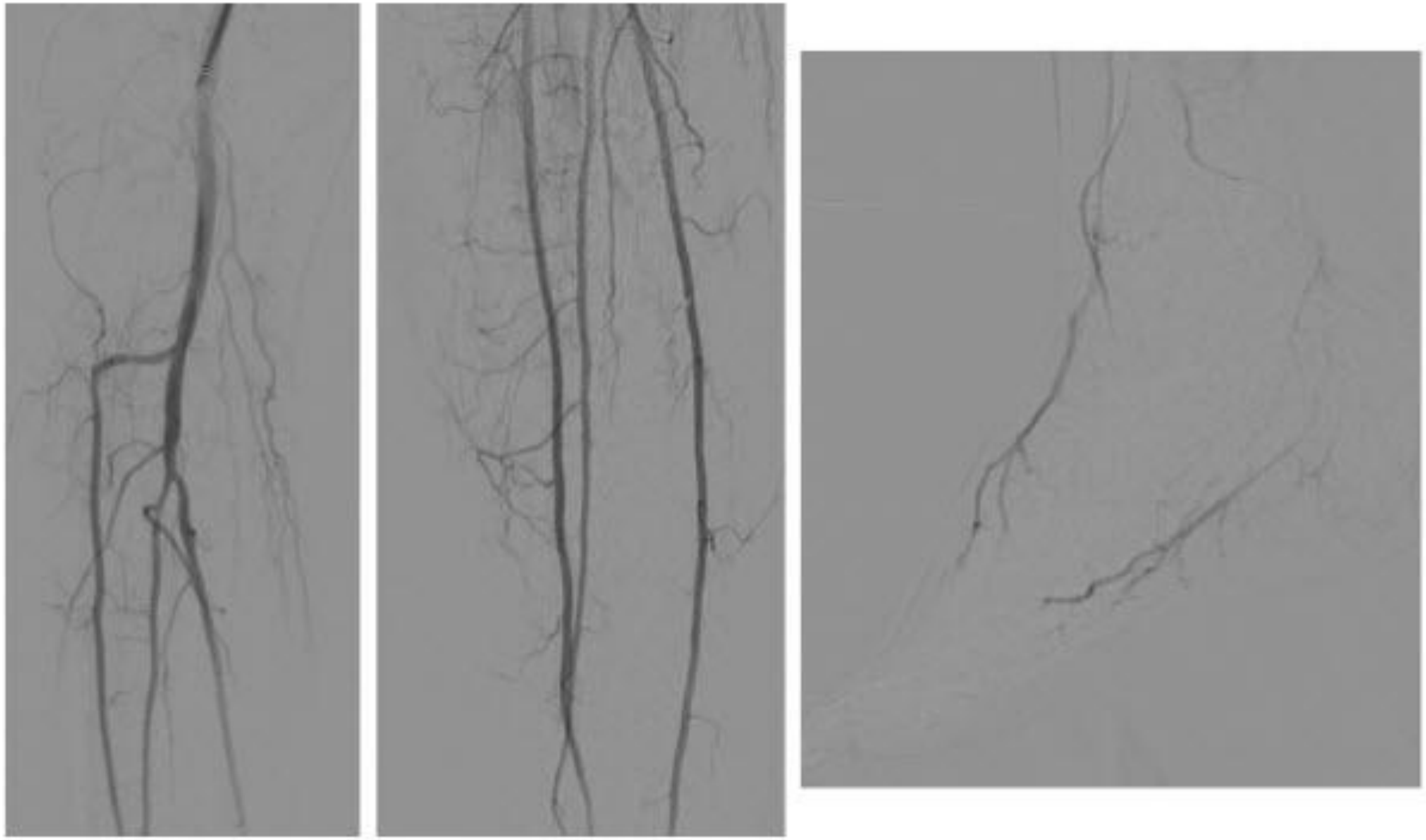

3c

Case 2

Patient presented with an occlusion of popliteal artery

Patient with trauma-associated occlusion beginning at the level of the popliteal artery. Screws can still be seen on the images. Final angiogram after thrombectomy:

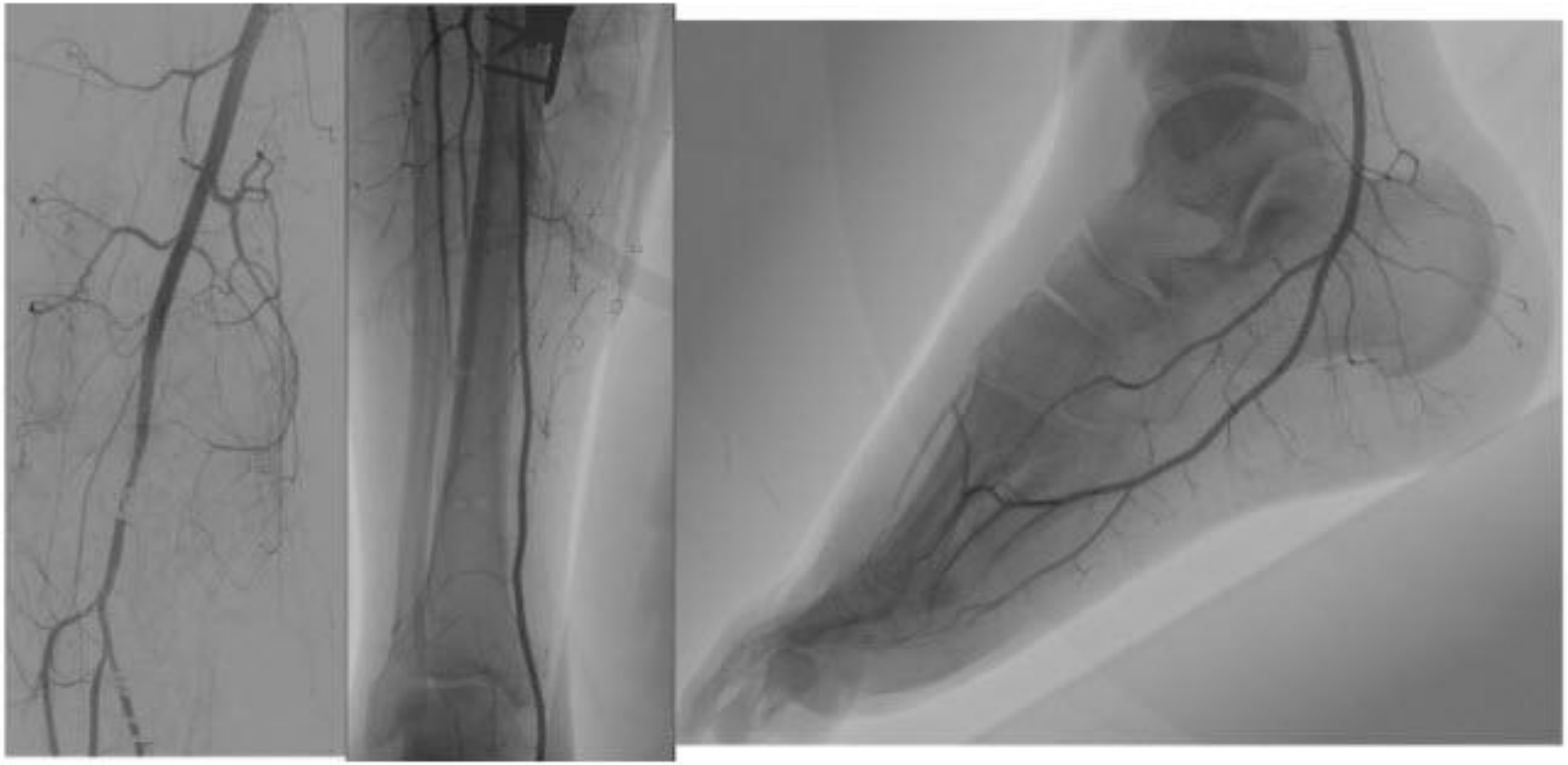

3a

Case 3

Patient with occlusion on the level of tibulofibular trunc

Images after thrombectomy

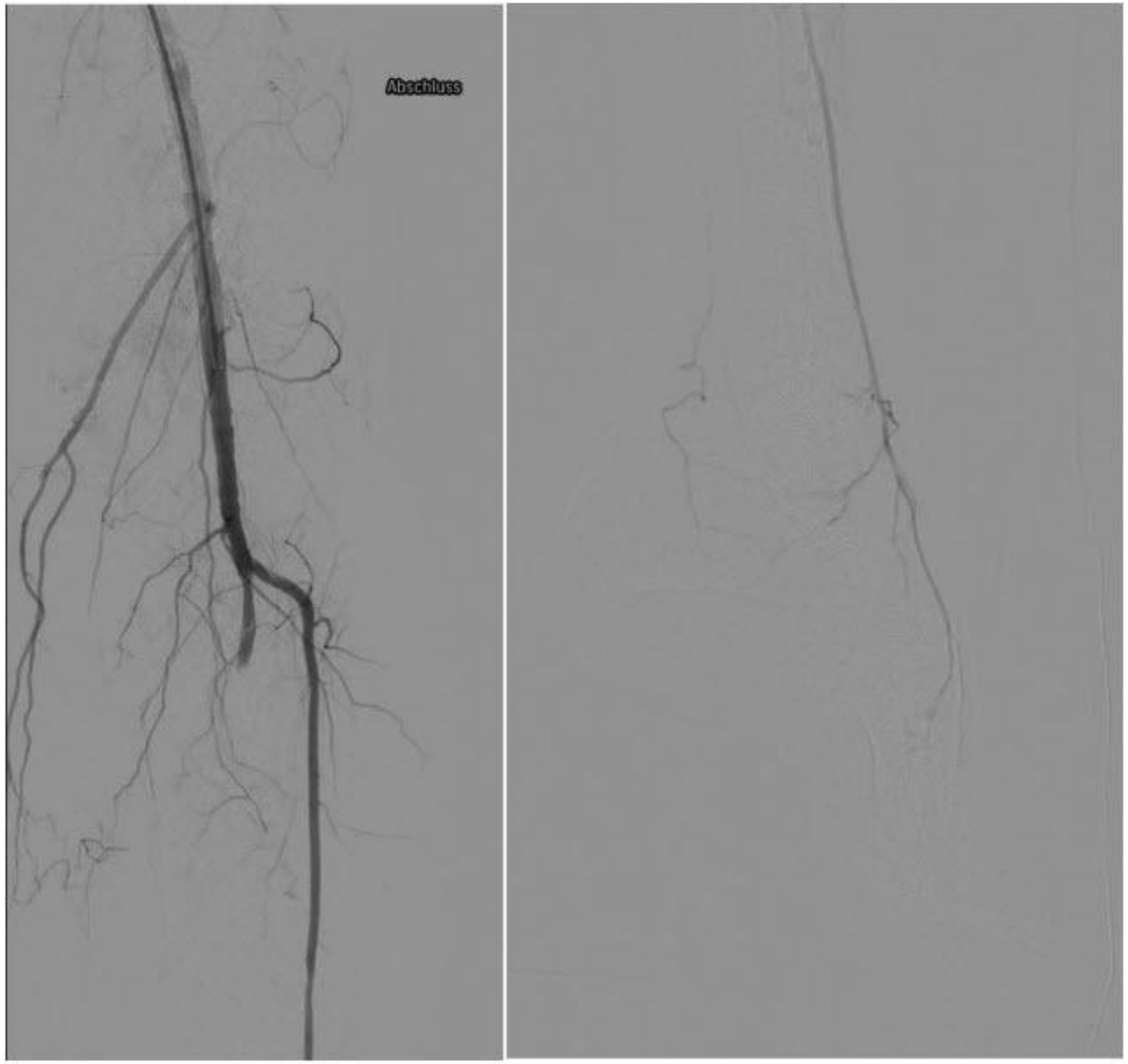

0

Case 4

Patient with inital occlusion of common femoral artery

Images after thrombectomy

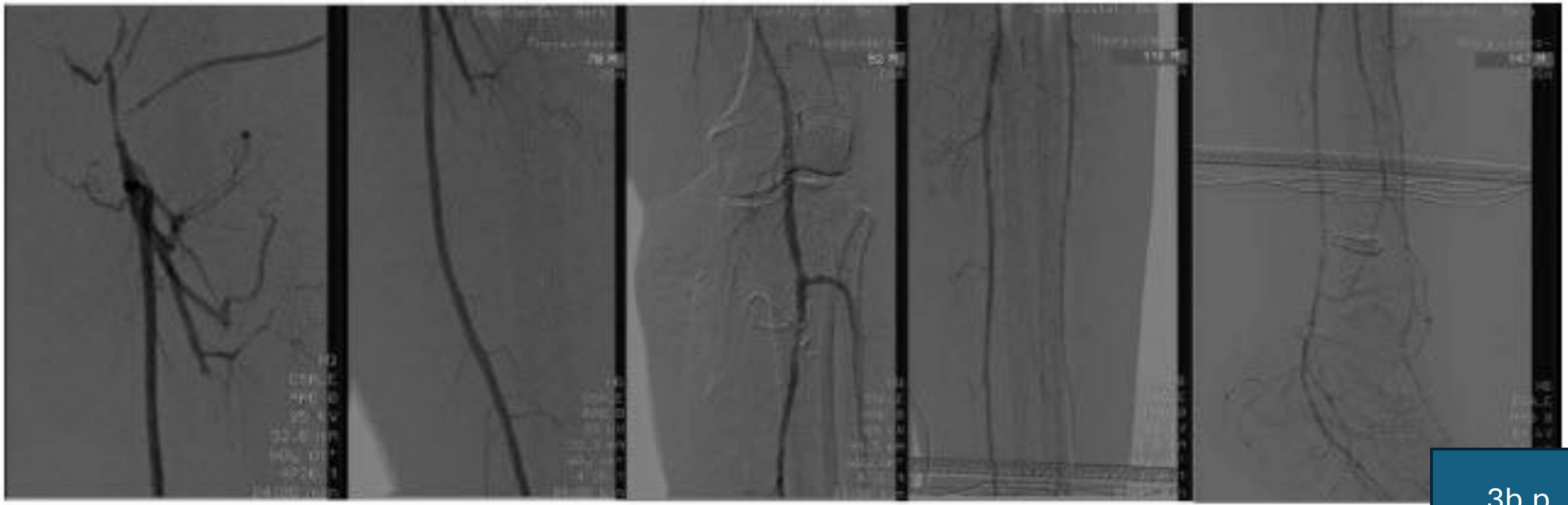

3b,p

Case 5

Patient with occlusion of common femoral artery

This angiogram was taken after a thrombectomy. The final image shows the anterior tibial artery, which extends to the upper ankle joint but does not reach the foot.

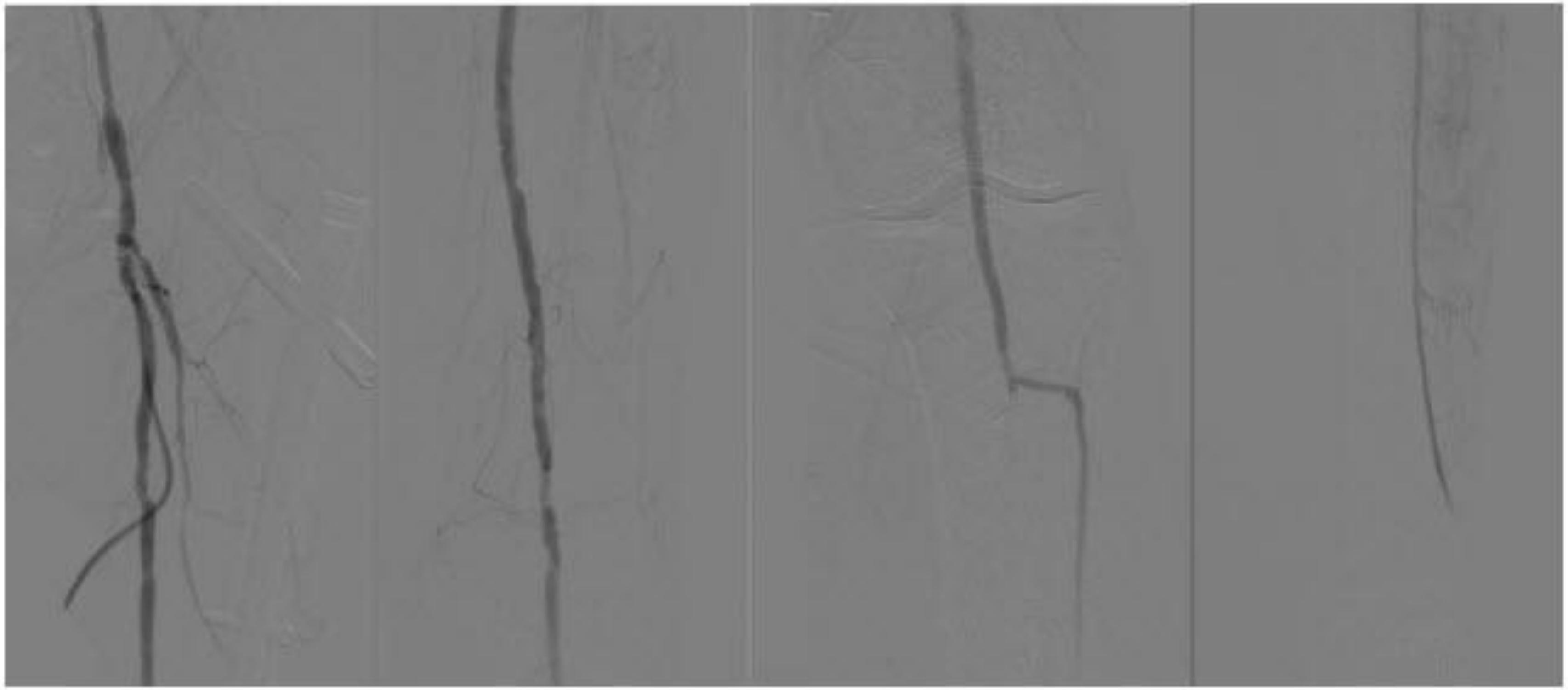

0

Case 6

Patient with initial occlusion of tibulofibular trunc

Images taken after thrombectomy. The second image is a later version to the first series due to the slow flow of the contrast medium it could not be captured in a single picture.

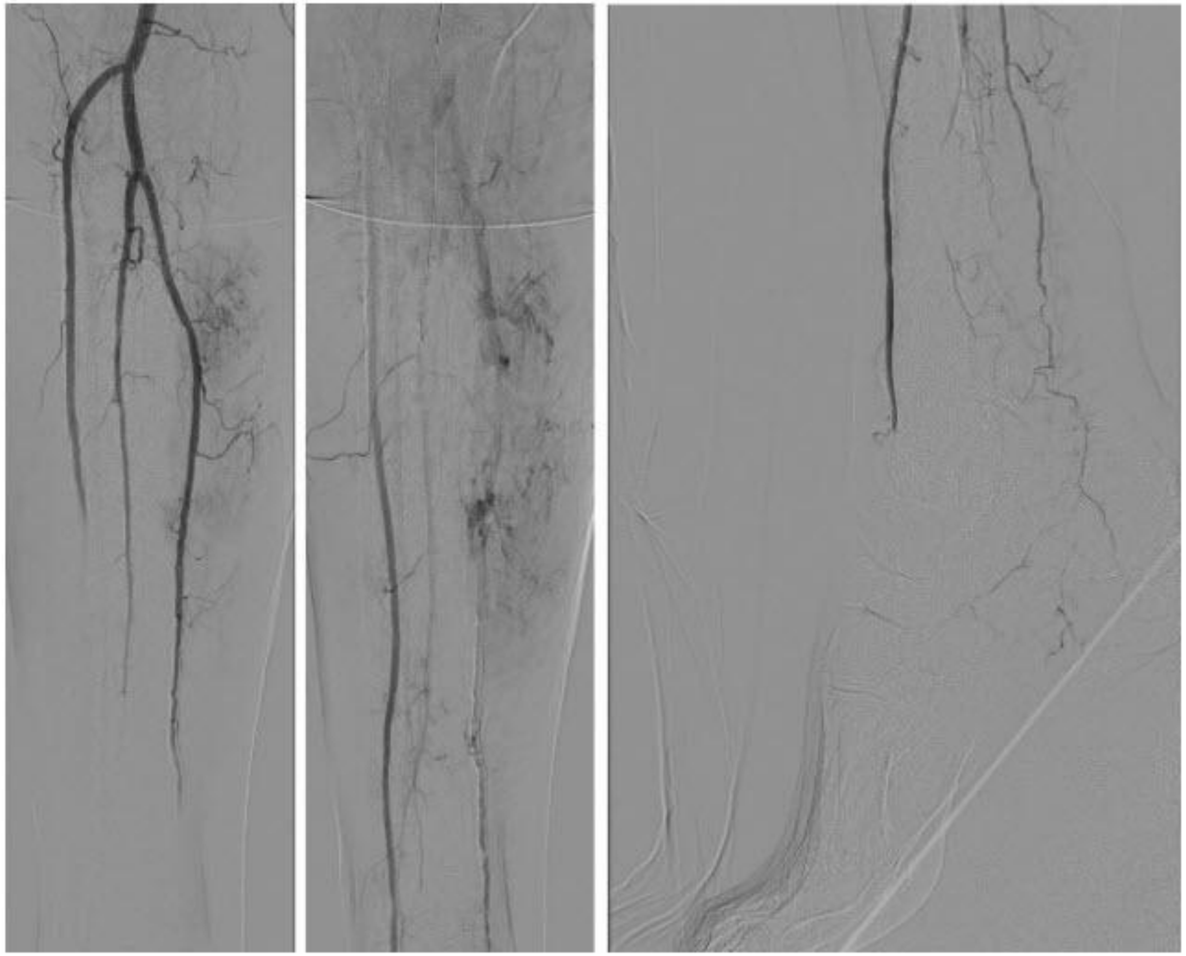

Not applicable

Case 7

Patient after thrombectomy of tibulo-fibular trunc

Images after thrombectomy. Second image is again the later filling of the angiogram.

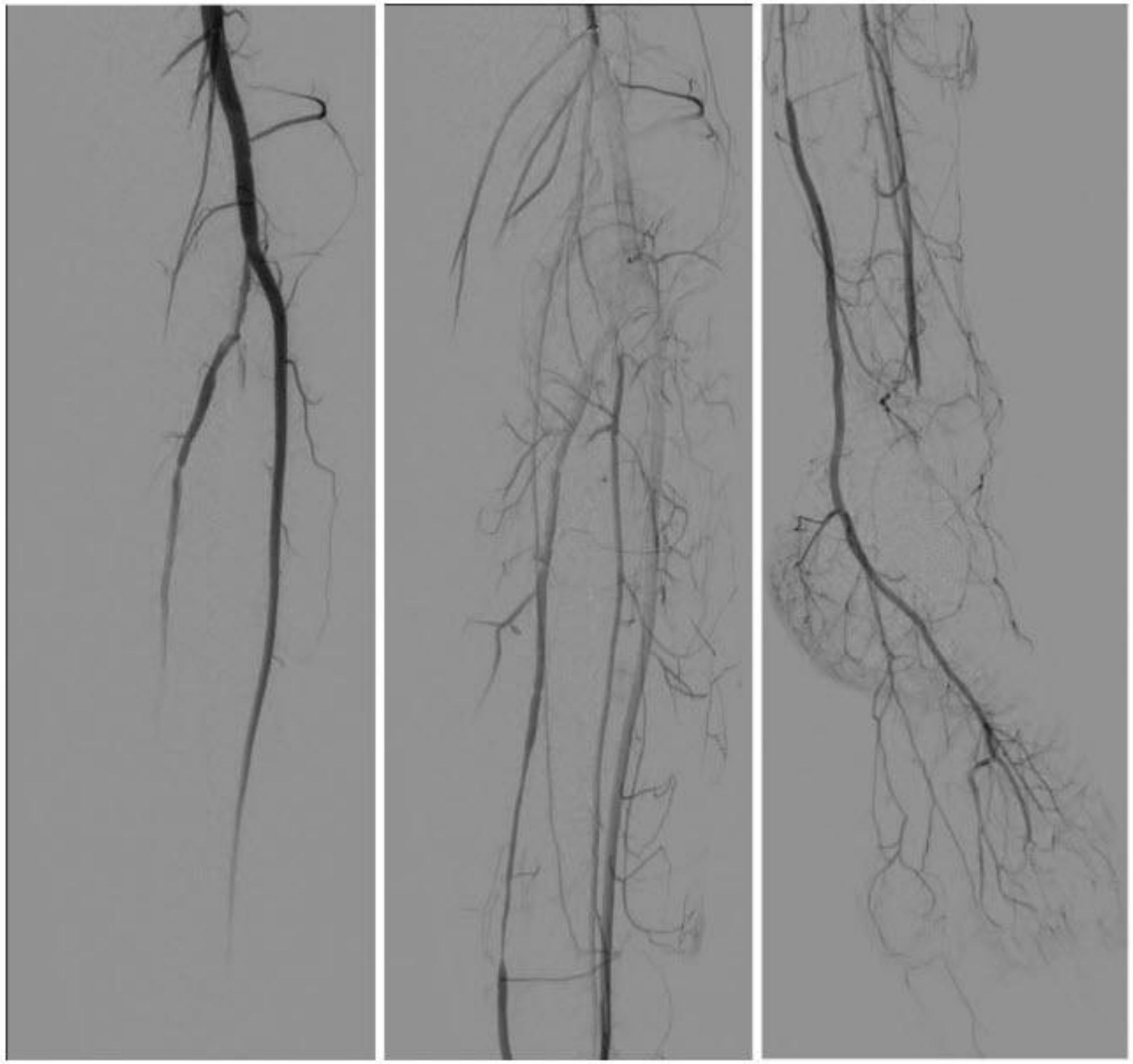

2b

Case 8

Patient after occlusion of external iliac artery

Images after thrombectomy at the level of the common femoral artery and the tibiofibular trunk. The third image shows the later filling of the second image (the superficial femoral artery).

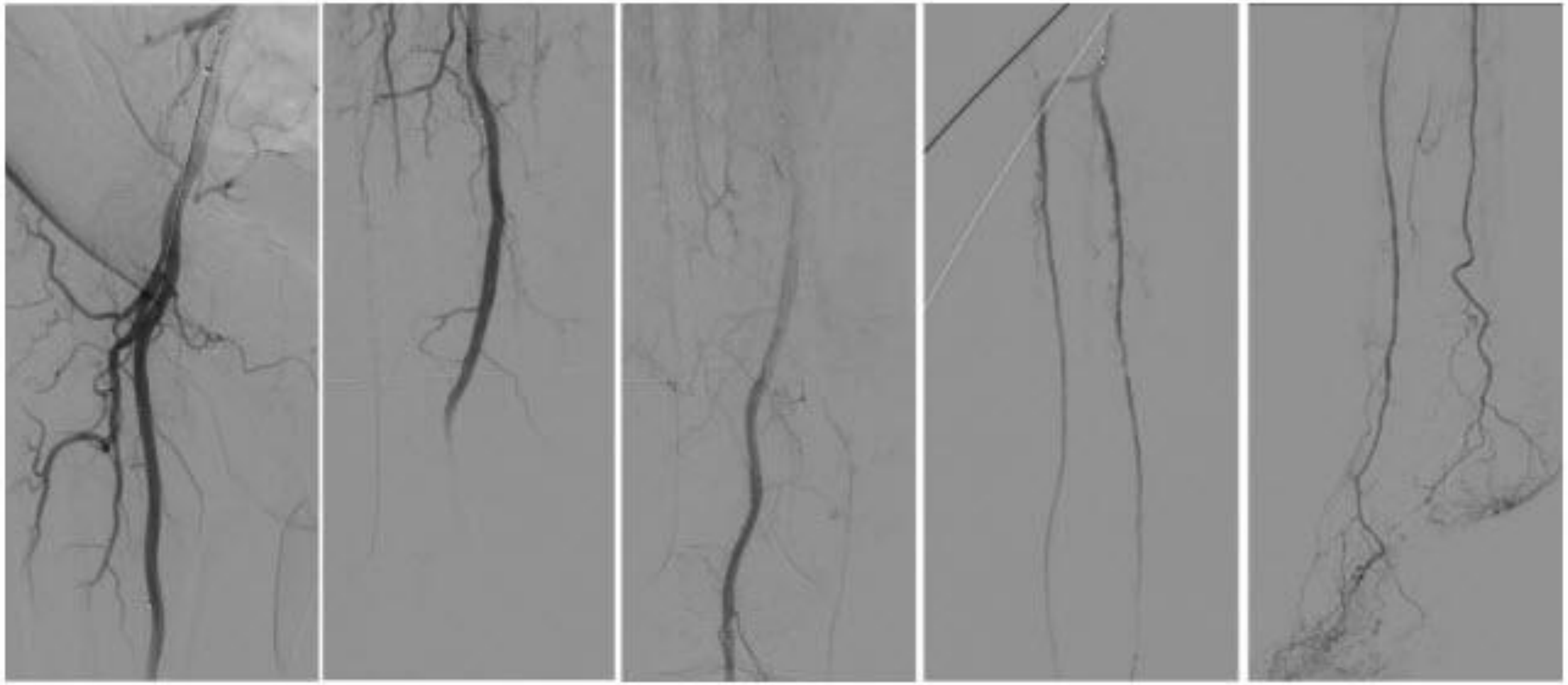

3b

Case 9

Patient with initially occlusion of tibulofibular trunc

Angiogram after thrombectomy, second image is a late filling of the first one.

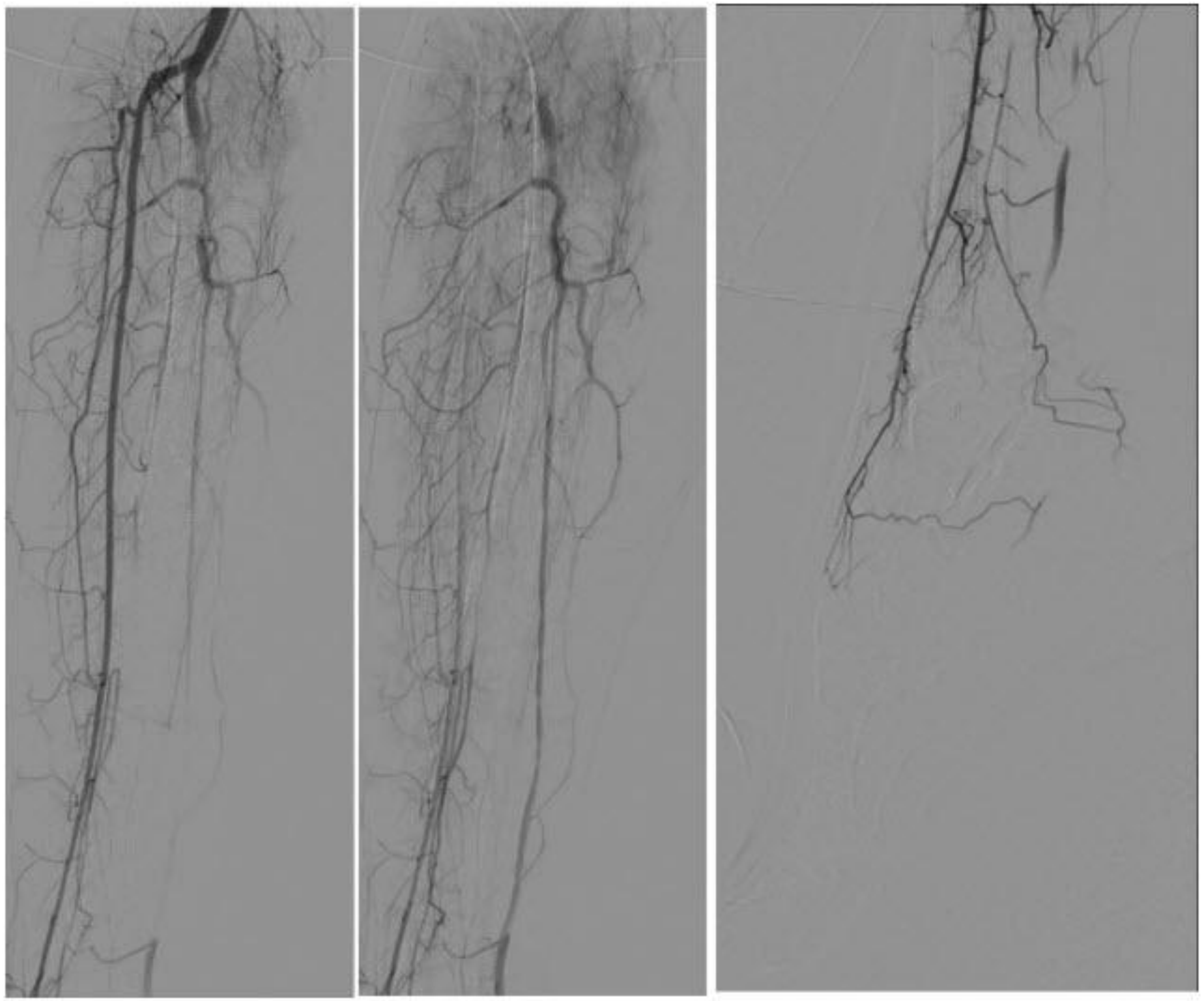

Case 10 (last one!)

Patient of occlusion of distal popliteal artery

Images after thrombectomy. Second image is a later filling of the first one, as well as the image of the foot (anterio-posterior view): fourth image is the later filling ot the trird one.

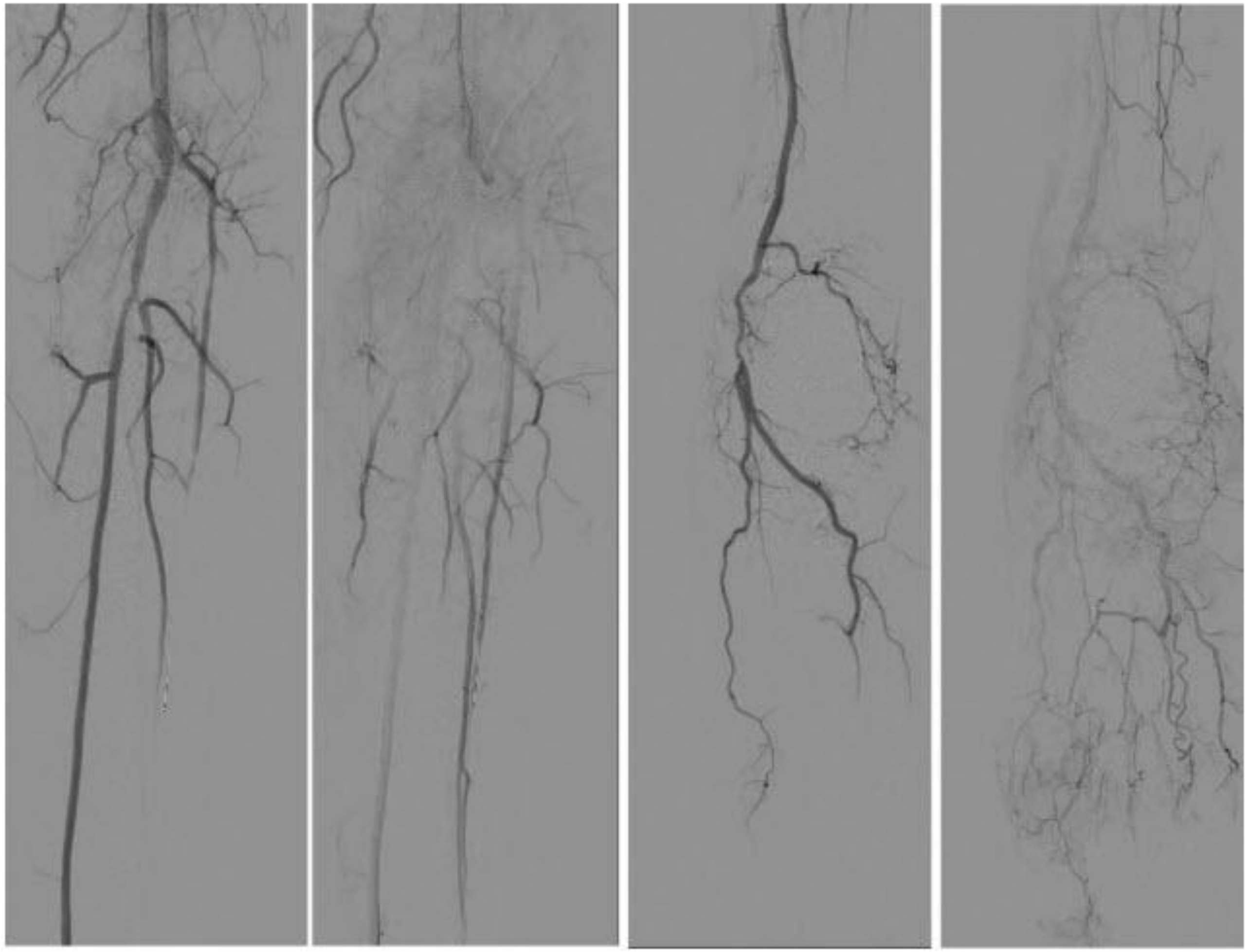

2b
