# Supplementary material for: The thrombectomy in limb ischemia score (TILI-Score): score proposal and results of an interobserver readability survey
Source: Int J Cardiovasc Imaging. 2026 Feb 18;42(4):711–20. doi: 10.1007/s10554-026-03617-9 (PMC13053501; doi:10.1007/s10554-026-03617-9)
Supplement: Supplementary file 2 — Supplementary Material 2 [file 10554_2026_3617_MOESM2_ESM.pdf]

Expert Number (random)	Total passive and active experience (working in vascular center, attending vascular conferences, being trained and perform the vascular interventions)	Active expertise (years of training in vsascular interventions)	Estimated case load per year in the last year
1	7	3	100
2	6	2	100
3	10	7	100
4	22	22	200
5	5	5	120
6	16	7	200
7	7	5	70
8	5	1	150
9	15	15	100
10	20	15	200
**Mean**	**11.3**	**8.2**	**134**
