# Supplementary material for: The thrombectomy in limb ischemia score (TILI-Score): score proposal and results of an interobserver readability survey
Source: Int J Cardiovasc Imaging. 2026 Feb 18;42(4):711–20. doi: 10.1007/s10554-026-03617-9 (PMC13053501; doi:10.1007/s10554-026-03617-9)
Supplement: Supplementary file 3 — Supplementary Material 3 [file 10554_2026_3617_MOESM3_ESM.pdf]

observer	1 Main TILI Grade is:	1 Periphe	2 Main TI	2 Peripheral emb	3 Main T	3 Peripheral en	4 Main TI
1	3	c	3	a	0	not applicable	3
2	3	c	3	a	0	not applicable	3
3	3	c	2	c	0	not applicable	2
4	3	c	3	a	0	not applicable	3
5	3	c	3	a	0	not applicable	3
6	3	c	0	not applicable	0	not applicable	3
7	3	b	3	a	0	not applicable	2
8	3	c	3	a	0	not applicable	2
9	3	b	3	a	0	not applicable	2

4 Perip	5 Main	5 Periphera	6 Main TIl	6 Periphe	7 Main TILI G	7 Peripheral	8 Main TILI	8Periphera	9 Main TI
b	0	not applical	3	a	2	a	3	b	0
c	0	not applical	3	b	3	b	3	b	2
c	2	a	3	a	3	a	2	b	2
c	3	not applical	3	a	2	b	3	b	2
b	0	not applical	not applic	not applic	2	b	3	b	1
b	2	not applical	3	b	2	a	3	b	2
b	0	not applical	not applic	not applic	2	a	3	b	1
b	2	not applical	3	a	2	a	3	b	1
b	0	not applical	2	a	2	a	3	b	1

9 Peripheral	10 Main TILI	10 Peripheral embolisation score is
not applicabl	3	a
a	3	a
a	2	a
a	2	b
not applicabl	3	b
a	3	a
not applicabl	2	a
not applicabl	3	a
not applicabl	2	b
